# Comprehensive analysis of three *TYK2* gene variants in the susceptibility to Chagas disease infection and cardiomyopathy

**DOI:** 10.1371/journal.pone.0190591

**Published:** 2018-01-05

**Authors:** Daniel A. Leon Rodriguez, Marialbert Acosta-Herrera, F. David Carmona, Nuria Dolade, Sofia Vargas, Luis Eduardo Echeverría, Clara Isabel González, Javier Martin

**Affiliations:** 1 Instituto de Parasitología y Biomedicina ‘López-Neyra’, IPBLN-CSIC, PTS Granada, Granada, Spain; 2 Grupo de Inmunología y Epidemiología Molecular, GIEM, Facultad de Salud, Universidad Industrial de Santander, Bucaramanga, Colombia; 3 Departamento de Genética e Instituto de Biotecnología, Universidad de Granada, Granada, Spain; 4 Clínica de Falla Cardíaca y Trasplante. Fundación Cardiovascular de Colombia. Floridablanca, Santander, Colombia; Universidade Federal de Minas Gerais, BRAZIL

## Abstract

Tyrosine kinase 2 (TYK2) is a member of the Janus kinases family implicated in the signal transduction of type I interferons and several interleukins. It has been described that genetic mutations within *TYK2* lead to multiple deleterious effects in the immune response. In this work, we have analyzed three functional independent variants from the frequency spectrum on the *TYK2* gene (common and low-frequency variants) suggested to reduce the function of the gene in mediating cytokine signaling and the susceptibility to infections by *Trypanosoma cruzi* and/or the development of Chagas cardiomyopathy in the Colombian population. A total of 1,323 individuals from a Colombian endemic region for Chagas disease were enrolled in the study. They were classified as seronegative (n = 445), seropositive asymptomatic (n = 336), and chronic Chagas Cardiomyopathy subjects (n = 542). DNA samples were genotyped using TaqMan probes. Our results showed no statistically significant differences between the allelic frequencies of the three analyzed variants when seropositive and seronegative individuals were compared, therefore these variants were not associated with susceptibility to Chagas disease. Moreover, when Chagas cardiomyopathy patients were compared to asymptomatic patients, no significant associations were found. Previous reports highlighted the association of this gene in immune-related disorders under an autoimmunity context, but not predisposing patients to infectious diseases, which is consistent with our findings. Therefore, according to our results, *TYK2* gene variants do not seem to play an important role in Chagas disease susceptibility and/or chronic Chagas cardiomyopathy.

## Introduction

According to the World Health Organization (http://www.who.int/chagas/en/) and the Center for Disease Control and Prevention (https://www.cdc.gov/parasites/chagas/), 8 million people suffer from Chagas disease worldwide, mainly in Latin America. This disorder is caused by the infection with the protozoan parasite *Trypanosoma cruzi* [1, 2]. Chagas disease has two clearly identified stages, *i*.*e*. an early acute phase and a chronic phase, and approximately 30% of infected individuals develop cardiac and/or digestive complications [1]. It is believed that host genetic factors could be implicated in the development of these complications, as it has been observed in other infectious diseases [3]. During the last years, several studies have been carried out in Chagas endemic populations, reporting evidences of a genetic component influencing the susceptibility to infection by *T*. *cruzi* and/or chronic Chagas cardiomyopathy [4].

The innate immune response to *T*. *cruzi* starts immediately after infection by controlling the replication and spread of the parasite in the host tissues. In this phase, several cytokines including interleukin 12 (IL-12), tumor necrosis factor alpha (TNF-α), interferon gamma (IFN-γ) and reactive nitrogen species, regulate parasite invasion. Subsequently, an adaptive response characterized by a strong and persistent Th1 response dependent of IL-12 takes place, which facilitate the parasite clearing in the host [5]. Some studies suggest that an uncontrolled inflammatory response may originate chronic complications [5–7]. However, other studies point to the possible role of parasite persistence to originate such complications [7, 8]. From a genetic point of view, different genes of the inflammatory immune response have shown statistically significant association with a differential risk to infection and to the development of chronic cardiomyopathy. Therefore, it seems that the inflammatory immune response is the clue for controlling infection in early stages and to avoid the appearance of more severe symptoms. Altogether, the evidence indicates that parasite persistence is fundamental for the onset of chronic Chagas cardiomyopathy.

The tyrosine kinase 2 (*TYK2*) gene encodes a member of the Janus kinases (JAK) family that is essential for signal transduction of type I interferons, and for the interleukins (IL) IL-6, IL-10, IL-12, and IL-13. Knockout mice for *Tyk2* displayed reduced responsiveness to type I interferons, lowered production of IFN-γ in spleen cells after treatment with IL-12 and IL-18, altered production of NO, increased susceptibility to viral infection, and altered differentiation into Th1 cells from naïve helper T cells [9, 10]. Moreover, it has been described in a previous report that the minor allele of some genetic variants mapping to this *locus* were able to reduce the function of the gene in mediating cytokine signaling *in vitro* and in a humanized mouse model [11]. In humans, patients with genetic mutations on *TYK2* exhibited multiple deleterious effects in the immune response, including impaired responses to IL-12 and type I interferons, increased susceptibility to microorganisms (virus, fungi and mycobacteria), and an imbalanced Th1/Th2 differentiation [12, 13]. Therefore, and based on all the above, we decided to perform a genetic analysis of the TYK2 gene in order to evaluate its possible influence in the susceptibility to infection by *T*. *cruzi* and/or the development of Chagas cardiomyopathy.

## Materials and methods

### Study subjects

For this study, 1,323 Colombian individuals from the endemic area of Santander State, Colombia, were enrolled. According to the National Health Institute of Colombia, the prevalence of Chagas disease in this region ranges between 1.44% and 2.50%. The participants of this study were selected either after a medical visit to the endemic area by the local hospitals or after a visit to the “Fundación Cardiovascular de Colombia". All participants were over 30 years old and did not belong to any specific ethnic group. In this regard, the population of this region of Colombia is mainly composed of mestizo individuals, without any specific ancestry.

First, the study group was classified according to the results of two serological tests (enzyme-linked immunosorbent assay and a commercial indirect hemagglutination test) as seropositive individuals (n = 878) and seronegative individuals (n = 445), using this last subgroup as controls. The seropositive individuals were clinically evaluated by electrocardiograms and echocardiograms and subdivided into chronic Chagas cardiomyopathy patients (n = 542) and asymptomatic individuals (n = 336). The mean age of participants was 51.77 years for seronegative individuals, 53.41 for asymptomatic individuals and 61.13 for chronic Chagas cardiomyopathy patients. The sex distribution for the entire group was 56% female and 44% male.

### Ethics statement

The Ethics Committees from the Universidad Industrial de Santander and Fundación Cardiovascular de Colombia approved this study in accordance with the ethical standards laid down in the 1964 Declaration of Helsinki. Written informed consent was obtained from all subjects prior to participation.

### SNP selection and genotyping

In order to analyze the possible role of *TYK2* on the genetic susceptibility to Chagas disease, we followed a candidate gene strategy. We analyze three missense independent variants (0.001≥*r*^*2*^≥0.19): two low-frequency variants (rs34536443 and rs12720356), suggested to reduce the function of the gene in mediating cytokine signaling [11], and one common missense variant (rs2304256). This SNP selection was based on their functional implication in signal transduction and their association with other immune-mediated conditions, such as rheumatoid arthritis, systemic lupus erythematosus and systemic sclerosis [14–17], given that a disturbance on the immunological balance could potentially lead to several and somehow related disease states [11, 18].

Genomic DNA was isolated from 5–10 ml of EDTA anticoagulated whole blood using standard salting-out techniques. The three SNPs were genotyped using TaqMan allelic discrimination assays from Thermo Fisher Scientific (Waltham, Massachussetts, USA. The genotyping was performed on a LightCycler 480 real-time PCR system (Roche Diagnostics, Basel, Switzerland).

### Statistical analysis

The Hardy-Weinberg equilibrium (HWE) was determined at a significance level of 0.01 for all groups of individuals. To test for association, we compared the allelic and genotypic frequencies between seronegative and seropositive individuals as well as between asymptomatic and chronic Chagas cardiomyopathy patients. Statistical significance for these comparisons was determined using the χ^2^ test and logistic regression analysis, when necessary. Odds ratios (OR) and 95% confidence intervals (CI) were calculated according to Woolf’s method. P-values lower than 0.05 were considered statistically significant. Pairwise linkage disequilibrium (LD; D’ and *r*^*2*^) and haplotypic blocks were estimated using an expectation–maximization algorithm as implemented in Haploview 4.2 [19]. Plink V1.07 (http://zzz.bwh.harvard.edu/plink/) [20] was used for all the analyses. The statistical power of our study was estimated with Power Calculator for Genetic Studies 2006 (CaTS; (http://www.sph.umich.edu/csg/abecasis/CaTS/) [21].

## Results

The three *TYK2* SNPs were in HWE in all the comparisons performed (P>0.01), and the genotyping success rate was over 95% for all analyzed genetic variants. In all cases, *r*^2^ values between each pair of SNPs were < 0.25, indicating independency amongst them.

In order to evaluate the association between *TYK2* gene variants and susceptibility to *T*. *cruzi* infection, the allelic frequencies of seronegative and seropositive individuals were compared (**Table 1**). For all tested SNPs, no statistically significant differences were observed in this comparison, thus suggesting that they do not influence the risk of infection by *T*. *cruzi*.

**Table 1:** Genotype and allele distribution for *TYK2* polymorphisms in seropositive and seronegative individuals.

			Genotype. N (%)				Allele test	
SNP	1|2	Group (N)	1|1	1|2	2|2	MAF %	P	OR [95% CI]
**rs34536443**	C|G	Seropositive (878)	1 (0.11)	34 (3.87)	843 (96.02)	2.05		
** **		Seronegative (455)	0 (0.00)	12 (2.64)	443 (97.36)	1.35	0.2013	1.53 [0.79–2.96]
**rs12720356**	C|A	Seropositive (877)	7 (0.80)	60 (6.84)	810 (92.36)	4.22		
** **		Seronegative (445)	1 (0.22)	38 (8.54)	406 (91.24)	4.49	0.7418	0.94 [0.63–1.39]
**rs2304256**	A|C	Seropositive (856)	37 (4.32)	272 (31.78)	547 (63.90)	20.21		
		Seronegative (440)	20 (4.55)	149 (33.86)	271 (61.59)	21.48	0.4504	0.93 [0.76–1.13]

On the other hand, we evaluated the possible association between *TYK2* genetic variants and chronic Chagas cardiomyopathy by comparing the allelic frequencies of the *TYK2* SNPs between chronic Chagas cardiomyopathy individuals and seropositive asymptomatic individuals. However, no statistically significant differences were observed for any of the analyzed genetic variants (**Table 2**).

**Table 2:** Genotype and allele distribution for *TYK2* polymorphisms in chronic Chagas cardiomyopathy (CCC) and asymptomatic individuals.

			Genotype. N (%)				Allele test	
SNP	1|2	Group (N)	1|1	1|2	2|2	MAF %	P	OR [95% CI]
**rs34536443**	C|G	CCC (542)	0 (0.00)	23 (4.24)	519 (95.76)	2.12		
** **		Asymptomatic (336)	1 (0.30)	11 (3.27)	324 (96.43)	1.94	0.7878	1.10 [0.55–2.18]
**rs12720356**	C|A	CCC (541)	5 (0.92)	33 (6.10)	503 (92.98)	3.97		
** **		Asymptomatic (336)	2 (0.60)	27 (8.04)	307 (91.37)	4.61	0.5175	0.86 [0.53–1.37]
**rs2304256**	A|C	CCC (530)	16 (3.02)	180 (33.96)	334 (63.02)	20.00		
** **		Asymptomatic (326)	21 (6.44)	92 (28.22)	213 (65.34)	20.55	0.7823	0.97 [0.76–1.23]

## Discussion

To our knowledge, this is the first study aimed to examine the role of functional genetic variants on *TYK2* in a parasitic disease. Specifically, we have analyzed common and low-frequency variants of the gene and their possible implication in the susceptibility to Chagas disease and its progression to Chagas cardiomyopathy. Our results suggest that *TYK2* does not seem to play an important role neither in the predisposition nor in the progression of an infectious condition like Chagas disease.

The influence of *TYK2* genetic variants have been previously assessed in other infectious diseases, such as tuberculosis [22] and in the human immunodeficiency virus (HIV) [18]. However, no significant associations have been described so far, which is consistent with our findings, even though the sample size of our study population provided an 80% statistical power to detect an OR = 2. Interestingly, the role of both rs34536443 and rs12720356 has been exhaustively analyzed by Dendrou and colleagues, in autoimmune diseases, other immune-mediated conditions [11]. The authors performed a meta-analysis including ten autoimmune diseases with further molecular, cellular, *in vivo* and structural follow-up studies. Although the rs12720356 variant showed inconsistent results among the different diseases, rs34536443*C was established as the lead independent signal conferring protection against all studied diseases, being able to impair the signaling of type I IFN, IL-12, and IL-23. However, this reduction on cytokine activity was not associated with immunodeficiency. Furthermore, individuals having the risk allele did not show an increase in hospitalization due to mycobacterial, bacterial, viral, or fungal infections. All this evidences indicate that rs34536443*C may allow a sufficient TYK2-mediated cytokine signaling to prevent immunodeficiency [11]. In light of this data, it is not surprising that a similar scenario may be plausible in infectious diseases, where individuals with this genotype may have a minimal signaling activity that helps with the immune response against the infection and, consequently, no differences in the outcome of the disease are related to these particular SNPs.

On the other hand, the rs2304256 represents a non-synonymous change (Val362Phe) within the FERM domain of the encoded protein which has been implicated in the interaction with transmembrane proteins such as cytokine receptors, binding the kinase domain and positively regulating its catalytic activity [23]. According to prediction tools [24, 25], the Val362Phe amino acid change is likely benign, thus indicating that it favors the protein activity.

In conclusion, given our results and previous evidences, the *TYK2* functional variants analyzed in this study are not likely important players in the risk of *T*. *cruzi* infection. However, we cannot rule out the possibility that a potential lack of sufficient statistical power may be limiting our results. In this work we show that none of the genetic variants are associated to Chagas disease susceptibility and/or progression to Chagas cardiomyopathy on infected individuals. Further studies in different populations may be necessary to confirm our findings and to serve as a basis for a better understanding of the genetic risk factors of Chagas disease.
